# Editorial: Immune Mechanisms Underlying the Increased Morbidity and Mortality of HIV-Exposed Uninfected (HEU) Children

**DOI:** 10.3389/fimmu.2017.01060

**Published:** 2017-09-06

**Authors:** Tessa Goetghebuer, Sarah L. Rowland-Jones, Tobias R. Kollmann

**Affiliations:** ^1^Université Libre de Bruxelles, Bruxelles, Belgium; ^2^Oxford University, Oxford, United Kingdom; ^3^University of British Columbia, Vancouver, BC, Canada

**Keywords:** HIV-exposed uninfected children, anti-retroviral therapy, HIV, public health policy, infection control

**Editorial on the Research Topic**

**Immune Mechanisms Underlying the Increased Morbidity and Mortality of HIV-Exposed Uninfected (HEU) Children**

Following the introduction of Prevention of mother to child transmission (PMTCT) strategies, the vast majority of infants born to HIV-infected mothers worldwide are not infected at birth. For reasons that remain unexplained, HIV-exposed uninfected (HEU) infants are at increased risk of suffering from poor health particularly during the early years of life, including increased susceptibility to infectious diseases, growth retardation, and delayed neurological development. This population is often forgotten, despite accounting for a large proportion of all births in sub-Saharan Africa (up to 30% of newborn children in southern Africa). For example, there are currently no guidelines for medical care to these children, and follow up and clinical care varies from one country to another. Even at international HIV or pediatric meetings, the plight of the HEU infants is rarely mentioned, despite the fact that these infants represent the next wave of the HIV epidemic.

The objective of this Research Topic is to present as complete a picture as is currently possible of the particular phenotype (clinical and biological) of HEU infants in the hope that this composite will begin to outline the origin/s of their suffering, i.e., the underlying mechanism/s.

The first publications reporting poor outcome of HEU children were from developing countries, with the large Zvitambo study demonstrating the increased morbidity and mortality of HEU compared to HIV-unexposed (HU) children. Evans et al. review the observations made in this cohort since 2006 and propose a framework to explain the susceptibility to infection and growth failure of HEU children, reviewing a number of key pathways that may drive immune activation and inflammation.

The increased risk of infections observed in HEU infants was described both before and after the availability of antiretroviral therapy (ART) for HIV prophylaxis. The review Slogrove et al. reports a systematic literature search of studies assessing the burden of infectious morbidity and mortality, and the patterns of infection in studies of HEU children both with and without comparison with an appropriate HU group.

Studies from both developed and developing countries have shown that HEU neonates are at increased risk of Group B streptococcal (GBS) sepsis. Dauby et al. review the possible explanations for this increased susceptibility, including maternal colonization, serotype strains, GBS maternal antibody transfer, and infant immune responses to GBS infection.

As reviewed by Ruck et al. while it is accepted that there is a contribution to the poor health outcomes of HEU infants from factors, such as increased exposure to pathogens from sick household contacts, poor socioeconomic conditions, replacement feeding, and exposure to ante- and postnatal prophylactic ART, exposure *in utero* to an immune environment perturbed by HIV infection could on its own lead to increased susceptibility to infection. Along the same lines, the potential for an altered transfer of maternal immune factors to affect the ontogeny of adaptive and innate immunity in HEU infants is discussed by Abu-Raya et al.; Slogrove et al.; Maloupazoa Siawaya et al.

The potential involvement of early CMV infection transmitted from HIV+ mothers with CMV reactivation during pregnancy in the impaired growth, neurological development, and immune responses of HEU infants is discussed by Filteau and Rowland-Jones.

As illustrated in the paper by Thorne and Tookey, the comprehensive clinic-based follow-up (and particularly long term follow-up) of HEU children is difficult even in most European countries. In the United States, the Pediatric HIV/AIDS Cohort Study (PHACS) is a multicenter cohort aiming to monitor the health of HEU children and to determine the safety of *in utero* exposure to ART. Van Dyke et al. present the trigger-based design of the Surveillance Monitoring for ART Toxicities (SMARTT) study, with reassuring results related to *in utero* ART exposure to most of the anti-retroviral drugs.

During the first HEU workshop held in Vancouver in 2015, hosted by the University of British Columbia, it became clear that there now exists a community of clinicians, epidemiologists, and basic scientists focused on deciphering the underlying causes for the increased morbidity observed in HEU infants, and aiming to advocate globally for an urgent increase in attention paid to this destructive wave of the HIV epidemic. A second HEU workshop took place in KwaZulu-Natal in 2016, where Slogrove et al. identified several concrete issues that should be overcome in order to move forward to harmonize outcome measurement within cohorts, together with improved surveillance strategies. The aim of these workshops was to place the HEU child in a more prominent position on the global HIV research and Public Health agenda, and will be followed by a third workshop in Paris in July 2017.

This rapidly growing, highly vulnerable population, long identified as requiring special attention, is now finally beginning to receive the interest it deserves from the scientific community. Clearly, more research still is needed to better understand the pathogenetic mechanisms underlying the poor health of HEU children and, eventually, to develop interventions that improve their outcomes and promote their good health. However, the manuscripts included in this Research Topic begin to provide insights across very different settings worldwide that together point toward actions needed to improve the health of HEU infants. Specifically:

1. Establish clinical cohorts of sufficient size to collect relevant data and biological samples from HEU and HU controls that permit rigorous interrogation of cause and effect, and harmonize these efforts across cohorts around the world.
2. Alert national and international funding bodies to this growing crisis, with the explicit goal of securing the necessary funding for these studies.
3. Create an international consortium of experts to coordinate the above and to advocate in the name of HEU infants, who all too often belong to the marginalized, frequently voiceless sectors of our societies.

## Author Contributions

All authors listed have made a substantial, direct, and intellectual contribution to the work and approved it for publication.

## Conflict of Interest Statement

The authors declare that the research was conducted in the absence of any commercial or financial relationships that could be construed as a potential conflict of interest. The reviewer, TS, and handling editor declared their shared affiliation.

